# Latent Transformer Models for out-of-distribution detection

**DOI:** 10.1016/j.media.2023.102967

**Published:** 2023-12

**Authors:** Mark S. Graham, Petru-Daniel Tudosiu, Paul Wright, Walter Hugo Lopez Pinaya, Petteri Teikari, Ashay Patel, Jean-Marie U-King-Im, Yee H. Mah, James T. Teo, Hans Rolf Jäger, David Werring, Geraint Rees, Parashkev Nachev, Sebastien Ourselin, M. Jorge Cardoso

**Affiliations:** a Department of Biomedical Engineering, School of Biomedical Engineering & Imaging Sciences, King’s College London, London, UK; b RetiSpec, Toronto, Canada; c King’s College Hospital NHS Foundation Trust, Denmark Hill, London, UK; d Institute of Psychiatry, Psychology & Neuroscience, King’s College London, London, UK; e Institute of Neurology, University College London, London, UK; f Stroke Research Centre, UCL Queen Square Institute of Neurology, London, UK

**Keywords:** 41A05, 41A10, 65D05, 65D17, Transformers, Out-of-distribution detection, Segmentation, Uncertainty

## Abstract

Any clinically-deployed image-processing pipeline must be robust to the full range of inputs it may be presented with. One popular approach to this challenge is to develop predictive models that can provide a measure of their uncertainty. Another approach is to use generative modelling to quantify the likelihood of inputs. Inputs with a low enough likelihood are deemed to be out-of-distribution and are not presented to the downstream predictive model. In this work, we evaluate several approaches to segmentation with uncertainty for the task of segmenting bleeds in 3D CT of the head. We show that these models can fail catastrophically when operating in the far out-of-distribution domain, often providing predictions that are both highly confident and wrong. We propose to instead perform out-of-distribution detection using the Latent Transformer Model: a VQ-GAN is used to provide a highly compressed latent representation of the input volume, and a transformer is then used to estimate the likelihood of this compressed representation of the input. We demonstrate this approach can identify images that are both far- and near- out-of-distribution, as well as provide spatial maps that highlight the regions considered to be out-of-distribution. Furthermore, we find a strong relationship between an image’s likelihood and the quality of a model’s segmentation on it, demonstrating that this approach is viable for filtering out unsuitable images.

## Highlights

• We develop Latent Transformer Models for unsupervised out-of-distribution detection in 3D.
• This approach combines a VQ-VAE with a transformer for likelihood evaluation.
• Our method can successfully identify both far- and near-OOD data.
• The method further provides spatial maps to localise anomalies.
• Experiments on 2D computer vision datasets help further explain the success of our approach.

## 1 Introduction

An important aim of medical image analysis is to develop algorithms that can be deployed in clinical settings. These tools must be robust to the full range of potential inputs they might receive in a clinical context. There is increasing interest in the clinical deployment of deep learning algorithms, which require training on a dataset before deployment. We can expect clinical data to be much more diverse than the typically clean, sanitised datasets on which these algorithms are trained. Even if attempts are made to train on messier datasets with images exhibiting artefacts and other issues, we would expect that eventually the tool will be presented with data it has not seen during training. We can classify inputs as either near out-of-distribution (OOD), meaning images similar to the intended input but containing artefacts or corruptions, or far-OOD, such as images of the wrong modality of containing the wrong organ of interest. Deep learning algorithms perform well when operating in-distribution but can degrade unpredictably and substantially when operating on OOD data (Pooch et al., 2019, Stacke et al., 2019).

One approach to this problem is to incorporate measures of uncertainty into the task-specific model itself – such as a classification or segmentation network – enabling decisions to be referred to humans when they are presented with difficult or OOD data samples (Yang et al., 2021). The simplest approach uses the softmaxed probability of the predicted class as a measure of confidence (Hendrycks and Gimpel, 2016) or the size of the pre-softmax logits (Hendrycks et al., 2019). Bayesian Neural Networks (BNN), which learn a distribution of weights, are another alternative; one popular approach is to approximate a BNN using dropout-based variational inference (Gal and Ghahramani, 2016). Another common approach is to employ an ensemble of neural networks and use their degree of agreement as a measure of their certainty (Lakshminarayanan et al., 2017). It has been shown that incorporating example outliers during training can improve performance, but such methods have the significant downside of making assumptions on the form outliers will take (Hendrycks et al., 2018, Roy et al., 2022). A comprehensive evaluation of uncertainty methods for classification found that the quality of uncertainty measures degraded as the size of the distributional shift increased (Ovadia et al., 2019). Some work has evaluated these methods in the context of image segmentation but is typically confined to evaluating the utility of the uncertainty methods in-distribution (Jungo et al., 2018, Nair et al., 2020); work that does evaluate the uncertainty in an OOD setting typically investigates only small dataset shifts such as increased noise (Haas and Rabus, 2021) or lower quality scans (McClure et al., 2019).

A second approach is to filter out anomalous data before it is fed to the task-specific network, termed OOD detection. The most popular approach to OOD detection involves using a generative model that can quantify the probability that a data sample is drawn from the distribution on which the task-specific model was trained. These approaches are attractive for several reasons. They are fully unsupervised, requiring no additional labels or examples of OOD data. They are also decoupled from the downstream task, which allows them to be used as reusable blocks in complex pipelines that may run a number of different algorithms on the same data, potentially with different thresholds based on how sensitive each downstream task is to OOD data. Of the generative approaches, transformer-based architectures (Vaswani et al., 2017) are attractive for two reasons. Firstly, they allow for the computation of exact likelihoods, and secondly, they are proving highly effective general-purpose architectures, achieving state-of-the-art performance across a range of tasks in language (Devlin et al., 2018, Brown et al., 2020) and, increasingly, vision (Dosovitskiy et al., 2020, Zhai et al., 2022). The attention mechanism (Vaswani et al., 2017) has quadratic memory scaling with sequence length, making it computationally infeasible to use transformers to model the sequence of raw pixel values in high-dimensional medical images. A recent body of work has instead used transformers to model the compressed discrete latent space (Esser et al., 2021) of an image obtained from a vector-quantising model such as a VQ-VAE or VQ-GAN (Oord et al., 2017, Razavi et al., 2019). In this work, we term these Latent Transformer Models (LTM) in analogy to Latent Diffusion Models (LDM) that use a similar latent backbone to train diffusion models (Rombach et al., 2022, Sohl-Dickstein et al., 2015, Ho et al., 2020). LTMs have achieved state-of-the-art unsupervised pathology segmentation for 2D and 3D medical images (Pinaya et al., 2021, Pinaya et al., 2022) and can produce high-quality 3D generative images of the brain (Tudosiu et al., 2020, Tudosiu et al., 2022). These results suggest that LTMs might be applied to fully 3D OOD detection but, to our knowledge, no published work is attempting this.

We make three principal contributions in this work, focusing on the problem of segmentation of haemorrhagic lesions in head CT data. Firstly, we design a dataset containing both near- and far-OOD examples, allowing OOD detection methods to be comprehensively evaluated. Secondly, we use this dataset to examine combined task-and-uncertainty networks, evaluating segmentation uncertainty methods and demonstrating they can catastrophically fail, producing confidently wrong predictions. Finally, we apply LTMs to perform image-wide OOD detection on 3D images. We find LTMs can effectively flag OOD data that segmentation networks fail to perform well on, in both the near- and far-OOD scenarios, demonstrating their viability as a filter in clinical settings where robust and fully-automated segmentation pipelines are needed. This work extends the methodical and experimental details in Graham et al. (2021).

## 2 Methods

In this work, we focus on the challenge of segmenting Intracerebral Haemorrhages (ICH) in 3D head CT data. The following sections detail the development of OOD detections for model evaluation, the trained segmentation networks, and the approach to training the LTMs. Code for our method is available at https://github.com/marksgraham/transformer-ood.

### 2.1 Datasets

We use three datasets in this work; two head CT datasets (one used for training and an independent one for model evaluation), and a non-head CT dataset.

The **CROMIS** CT dataset contains 687 head CT scans, and was used for training all the models in this paper. All the CTs contain ICH and were acquired across multiple sites in the United Kingdom as part of a clinical trial (Werring, 2017, Wilson et al., 2018). Haemorrhage segmentation masks were drawn on 221 scans by an experienced researcher (PT).

**Fig. 1:**
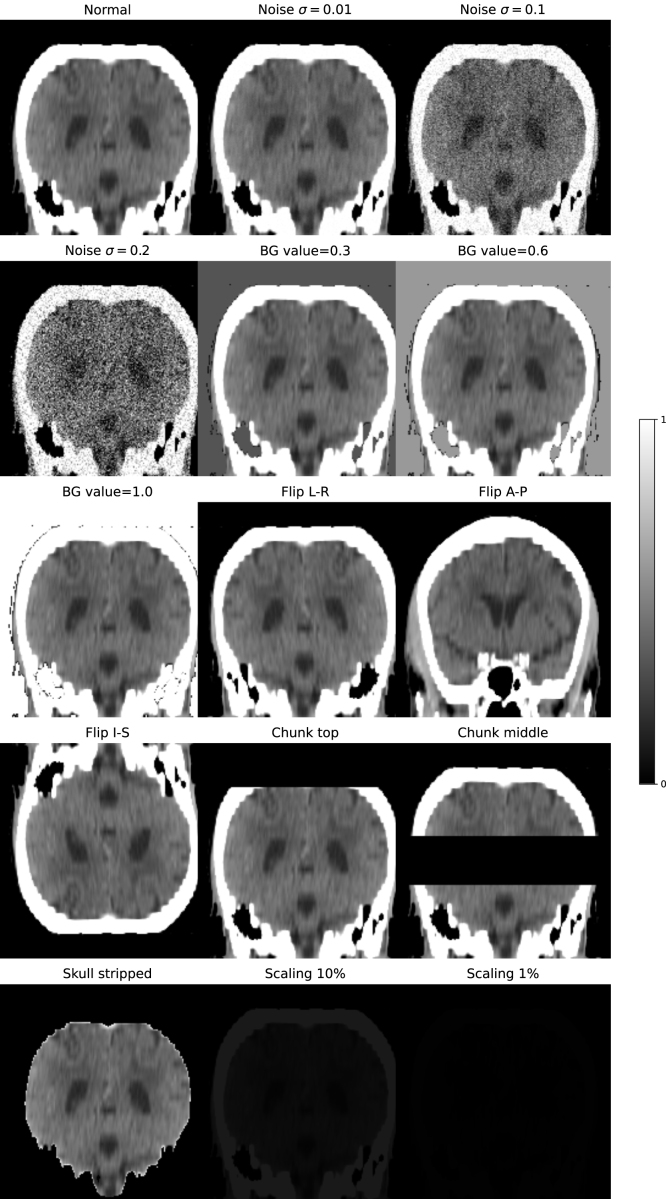
Example of all the corruptions applied to one subject from the KCH head CT dataset. All images are shown with the same intensity range. Corruptions are: Noise: Adding Gaussian noise $∼N\left(0,\sigma^{2}\right)$, BG value: replacing background value of 0 with a new constant value, Flip: invert image through described plane; Chunk: set a number of parallel slices = 0, Skull strip: remove skull, Scaling: reduce global image intensity by multiplying by a fixed factor.

The **KCH** CT dataset was used for algorithm validation. It consists of 47 clinical scans selected for the presence of ICH, all with ground-truth masks provided by an experienced neuroradiologist (JMU). This dataset was used to represent in-distribution test data, and it was further used to produce a set of corrupted scans to test our algorithms in the near-OOD setting. We applied a range of distortions and corruptions to each volume, designed to emulate a number of scenarios such as imaging artefact, image header errors, and errors in a preprocessing pipeline that may be run on volumes before data is input into a network. The corruptions included: addition of zero-mean Gaussian noise with three difference variances, σ∈{0.01,0.1,0.2}, inversion through each of the three central imaging planes (coronal, sagittal, and axial), removal of the skull using the method described in Muschelli et al. (2015), setting the image background to values not equal to 0, global multiplication of all image intensities by a fixed factor (either 0.1 or 0.01), and the deletion of a set of adjacent slices (or chunks) of the image (either in the central or upper portion of the scan). When the images were inverted, the same transformation was applied to the haemorrhage masks to ensure they remained aligned with the image. In total, 15 corruptions were applied to each image, creating a corrupted dataset of 705 images. Examples of the corruptions are shown in Fig. 1.

**Fig. 2:**
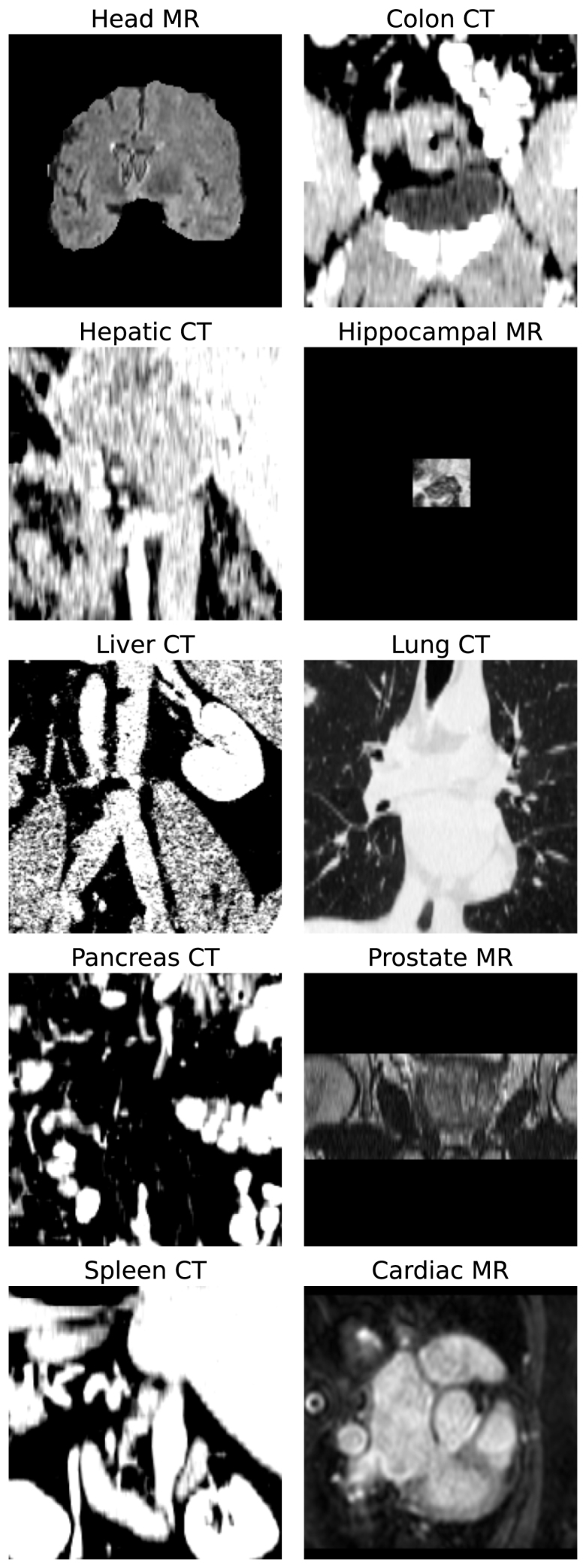
Example of images from the ten classes in the Medical Decathlon dataset, used as far-OOD data.

The **Medical Decathlon** dataset was used to test our algorithms in the far-OOD setting. The dataset consists of 3D medical images covering a variety of organs and imaging modalities, none of which are head CT. We selected 22 images (or as many available, if less than 22) from the test sets of each of the ten classes. A more detailed description of this dataset can be found in Simpson et al. (2019) and Antonelli et al. (2022). Examples of Medical Decathlon images are shown in Fig. 2.

Data processing was harmonised between all datasets as much as possible. All CT head images were registered to MNI space using an affine transformation, resampled to 1mm isotropic, tightly cropped to a 176 × 208 × 176 grid, intensities clamped between [−15,100] and then rescaled to lie in the range [0,1]. For the images in the Decathlon dataset, all were resampled to be 1mm isotropic and either cropped or zero-padded depending on size to produce a 176 × 208 × 176 grid. All CT images had their intensities clamped between [−15,100] and then rescaled to lie in the range [0,1], and all non-CT images were rescaled based on their minimum and maximum values to lie in the range [0,1].

### 2.2 Uncertainty-aware segmentation networks

In order to test methods that both perform a task and are able to flag OOD data, we implemented and tested three segmentation uncertainty methods commonly employed in the literature. The first was intended as a simple baseline and uses the softmax of the network’s output as a per-pixel probability map, and can be seen as the segmentation-based variant of the classification method described in Hendrycks and Gimpel (2016). The second method was an ensemble of N neural networks, identical in architecture, but each trained on a different subset of the data (Lakshminarayanan et al., 2017). We chose N = 5 based on recommendations from (Ovadia et al., 2019). The third method was an approximation of a BNN obtained through dropout-based variational inference, training each dropout layer with a dropout probability of p=0.5 and using 5 passes during inference to approximate the posterior (Gal and Ghahramani, 2016).

All networks used the same 3D UNet backbone (Ronneberger et al., 2015, Falk et al., 2019) as implemented in MONAI^1^ (Cardoso et al., 2022) as the ‘BasicUnet’ class, with (32, 32, 64, 128, 256) features in the 5 encoding layers, LeakyReLU activations and instance normalisation. Each network was trained using a batch size of 3 on 128 × 128 × 128 patches sampled from the volumes and augmented with both affine and elastic transformations. The Dice loss was used except for the baseline network, which was trained using cross-entropy loss as Dice is known to provide poorly calibrated, overly-confident predictions (Mehrtash et al., 2020). All networks were optimised using AMSGrad (Reddi et al., 2019) with a learning rate set to $1e^{-3}$, for a maximum of 300 epochs with early stopping if the validation loss did not improve for 60 epochs.

For each network, we sought to assign a single uncertainty value to each predicted lesion, defined as a single connected component. Firstly, we produced a per-voxel uncertainty map for each method. For the baseline method, this was the per-voxel softmax of the network output. For the remaining networks, we used the entropy between the N predictions at each voxel, $\left(1-\sum_{i}^{N}p_{i}\ln p_{i}\right)$ as described in Nair et al. (2020), where we subtracted the entropy from 1 so that larger values reflect higher certainty. We produce per-lesion certainty by taking the average of the per-voxel measures across each lesion, where each separate lesion is taken as each fully connected component from the majority vote prediction of each network.

### 2.3 Latent transformer models

**Fig. 3:**
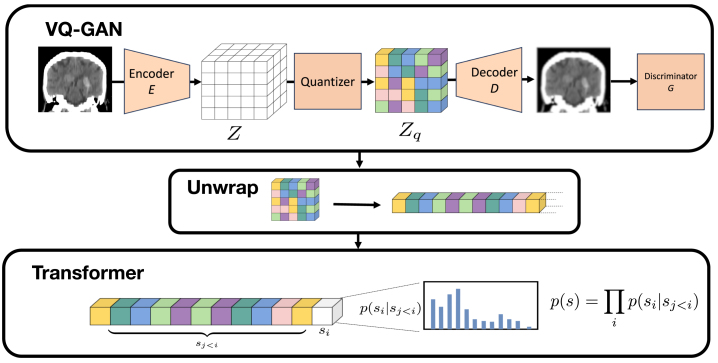
Overview of the LTM used for OOD detection. The VQ-GAN encodes the image and then quantises the encoding, according to a codebook of fixed size, to produce a discrete representation. This 3D representation is unwrapped to produce a 1D sequence, and a transformer is used to learn the distribution of these 1D sequences. Full details of the architecture are described in Section 2.3.

The LTM uses a VQ-GAN to compress the information content of each 3D volume into a discrete latent representation and a transformer to learn the probability density of these representations. An overview of the method is shown in Fig. 3.

The VQ-GAN (Esser et al., 2021) consists of an encoder E which takes input $x\in R^{H\times W\times D}$ and produces a latent representation $z\in R^{h\times w\times d\times n}$ where n is the dimension of the latent embedding vector. The representation is quantised by finding its nearest neighbour, as measured by an $L_{2}$ norm, in a ‘codebook’ containing K n-dimensional vectors. The representation is replaced with its nearest neighbour’s codebook index, k. A decoder D uses the quantised latent space to reconstruct the input, $\overset{ˆ}{x}\in R^{H\times W\times D}$. A discriminator G is used to differentiate between real and reconstructed images, encouraging the network to produce realistic reconstructions. Our implementation’s encoder contains four levels, each consisting of a convolution with stride = 2 and a residual layer, each followed by ReLU activations. This produces a latent space $2^{4}=16\times$ smaller along each dimension, so an input with size 176 × 208 × 176 is compressed to a latent size of 11×13×11=1573 elements. The codebook has K=256 elements, each with dimension n=256. The decoder also contained four levels, each consisting of a residual layer followed by a transposed convolution with stride = 2. The codebook was updated using the exponential moving average as described in Oord et al. (2017). The VQ-GAN paper combined the mean-squared error and a perceptual metric (Zhang et al., 2018) for the reconstruction loss — we used both these and an additional $L_{2}$ loss on the image’s Fourier representation (Dhariwal et al., 2020), as recommended in Tudosiu et al. (2020). Given state-of-the-art anomaly detection results have been reported in 2D using a simpler VQ-VAE with MSE loss (Pinaya et al., 2021), we also performed an ablation study to understand how the additional components of the VQ-GAN contributed to performance. Models were trained using the Adam optimiser (Kingma and Ba, 2014) with a learning rate = 1.65$e^{-4}$ and a batch size of 96 on an Nvidia DGX A100.

After training the VQ-GAN, we estimate the probability density of the training data’s latent space using a transformer. Each 3D discrete representation obtained from the trained VQ-GAN is flattened into a 1D sequence. The data-likelihood is represented as the product of conditional probabilities, $p\left(s\right)=\prod_{i}^{n}p\left(s_{i}|s_{<i}\right)$, with the transformer learning the distribution of $p\left(s_{i}|s_{<i}\right)$ by being trained to maximise the log-likelihood of the training data, where we use s rather than x to make clear we are computing the likelihood of the latent space sequence. In addition to estimating the likelihood p(s), we produced spatial likelihood maps by reshaping each $p\left(s_{i}|s_{<i}\right)$ from the 1D sequence back into the 3D shape of the latent representation and upsampling using nearest-neighbours (by a factor of 16 along each of the three image dimensions) to produce a spatial likelihood map of the same dimension as the input image. The transformer’s attention mechanism has a quadratic memory dependence on sequence length that makes it difficult to train on large sequences, even after significant compression with a VQ-GAN, so we made use of the more efficient Performer architecture (Choromanski et al., 2020), which uses a linearised approximation of the attention matrices to allow for training on longer sequences. We used a 22-layer Performer with 8 attention heads and a latent representation of size 256. The model was trained using the cross-entropy loss using a learning rate of $5e^{-4}$ and a batch size of 240 on an Nvidia DGX A100.

Both models were trained on the full CROMIS dataset. It should be noted that this dataset contains pathological images containing haemorrhages; the definition of in-distribution here is scans that are similar to the segmentation network’s training set, not scans that do not contain pathology, and the aim is to estimate whether a new input is similar enough to the segmentation network’s training set that the segmentation network will be able to perform suitably on it. This is distinct from anomaly segmentation works (Pinaya et al., 2021) where in-distribution is typically used to refer to data free of pathology.

**Fig. 4:**
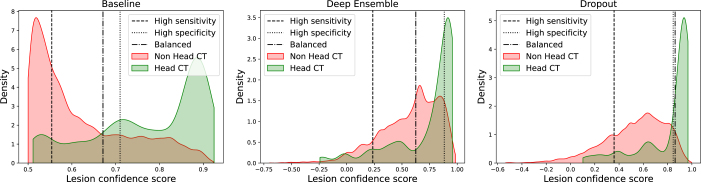
The distribution of per-lesion confidence scores for far-OOD non-head CT data and all true positive detections in the head CT dataset. Lines also show confidence thresholds for operating in three regimes: high-sensitivity (>90%), high-specificity (>90%) and balanced, with thresholds determined using the head CT dataset.

**Fig. 5:**
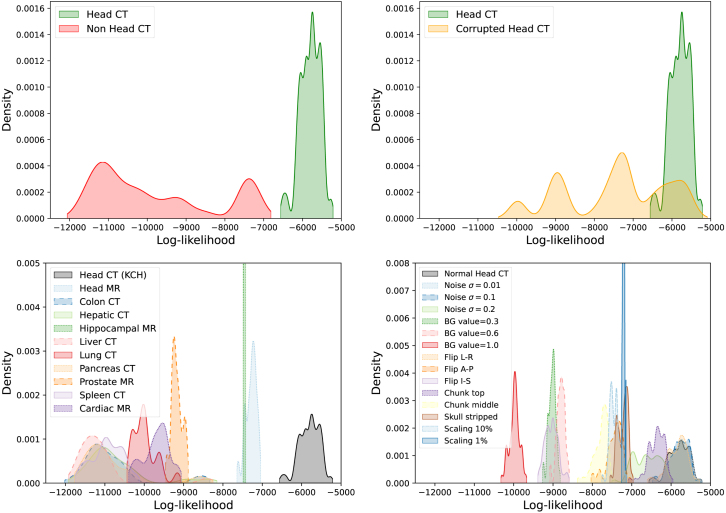
LTM log-likelihoods for far-OOD, near-OOD and in-distribution volumes. The top row shows coarse in-distribution vs. OOD plots, and the bottom rows show a finer breakdown by sub-class.

## 3 Results

### 3.1 Segmentation uncertainty

First, we examine the performance of segmentation algorithms in the far-OOD case, where images are of a different organ and/or modality than the intended target for segmentation. Any detection in this scenario is a false-positive (FP), and an ideal algorithm would either not predict the presence of lesions or would predict lesions with very low confidence. We calculated per-lesion confidence scores for each detection and compared them to the per-lesion scores of every true-positive (TP) detection from the normal head CT dataset. Fig. 4 shows that the distribution of lesion confidence scores for these two datasets overlap regardless of the segmentation uncertainty method used, meaning it is not possible to separate FP detections made on far-OOD data from TP detections on in-distribution data using any of these lesion confidence scores alone. The inability for these algorithms to perform sensibly in the far-OOD case motivates the need for explicit OOD detection models.

We also examined the performance of segmentation network uncertainty for near-OOD data, corrupted head CT scans. As these scans contain haemorrhages, the networks can make both TP and FP predictions. We defined a TP as a predicted lesion with at least 50% overlap with an overlapping GT lesion, and computed the AUC obtained when using the per-lesion certainty scores to classify lesions. Results are shown in Table 1. The networks are able to rule out FP lesions relatively well for certain types of corruption, including noise, image flipping, and the removal of ‘chunks’ from the data. However, the networks perform poorly for images with modified background values, or those that have been skull-stripped. For images with intensity scaling, all three methods report AUC ≤0.36, showing they tended to assign higher confidence scores to FP detections than they did to TP detections, a catastrophic failure for an uncertainty algorithm.

It is notable in Table 1 that the Baseline network reports higher AUC scores than the Deep Ensemble for data with no corruptions applied. This occurs because the baseline network makes numerous wrong predictions that it assigns high uncertainty to, which helps to inflate the AUC. This illustrates that AUC scores alone are not sufficient for assessing the effectiveness of segmentation networks. However, they suffice for making the key point here: none of the networks assessed are able to provide accurate measures of uncertainty when operating OOD.

**Table 1:** AUC for distinguishing TP and FP lesion detections using per-lesion confidence scores from several segmentation uncertainty methods, for near-OOD corrupted head CT data.

Network	Perturbation						
	None	Noise	Background	Flipping	Chunks	Skullstrip	Scaling
Baseline	0.84	0.82	0.38	0.86	0.87	0.45	0.33
Deep ensemble	0.75	0.85	0.42	0.77	0.82	0.54	0.36
Dropout	0.83	0.87	0.52	0.86	0.86	0.58	0.35

### 3.2 LTMs for OOD detection

We examined the ability of LTMs to filter out OOD inputs based on the image log-likelihood. Fig. 5 shows the distribution of log-likelihood values for far-OOD, near-OOD, and in-distribution classes, and AUC scores are reported in Table 2. The method provides perfect performance in the far-OOD case, with AUC = 1 for every sub-class. In the case of near-OOD data, classes on which the segmentation uncertainty performed poorly are distinguished well: images with adapted backgrounds, skull-stripping, and global intensity scaling are all distinguished with an AUC = 1. The method shows lower AUC for the two lower levels of noise applied (σ=0.1 and σ=0.01), though, as can be seen in Fig. 1, these noise levels are subtle. The method’s limited ability to distinguish L-R flipped data as OOD is not surprising, as these images exhibit L-R symmetry — in reality, OOD is not a binary label but a continuum.

**Table 2:** AUC for distinguishing between normal head CT and far- and near-OOD classes. Reported log-likelihood values are mean (std).

	Dataset	Log-likelihood	AUC
Non Head CT	Head MR	−7288 (134)	1.00
	Colon CT	−10 809 (789)	1.00
	Hepatic CT	−10 712 (763)	1.00
	Hippocampal MR	−7465 (20)	1.00
	Liver CT	−11 116 (658)	1.00
	Lung CT	−9957 (289)	1.00
	Pancreas CT	−10 798 (791)	1.00
	Prostate MR	−9140 (134)	1.00
	Spleen CT	−10 895 (382)	1.00
	Cardiac MR	−9661 (318)	1.00

Corrupted Head CT	Noise σ=0.01	−5796 (253)	0.49
	Noise σ=0.1	−5793 (237)	0.49
	Noise σ=0.2	−6637 (324)	0.98
	BG value = 0.3	−9022 (89)	1.00
	BG value = 0.6	−8803 (100)	1.00
	BG value = 1.0	−9979 (127)	1.00
	Flip L-R	−5850 (253)	0.55
	Flip A-P	−7435 (205)	1.00
	Flip I-S	−9036 (165)	1.00
	Chunk top	−6382 (214)	0.96
	Chunk middle	−7784 (179)	1.00
	Skull stripped	−7226 (125)	1.00
	Scaling 10%	−7436 (119)	1.00
	Scaling 1%	−7205 (25)	1.00

Normal Head CT		−5803 (256)	–

These subtler corruptions (noise and L-R flipping) are classes for which the segmentation network uncertainty measures perform well, suggesting these corruptions are more in-distribution and explaining why they have been assigned likelihoods more similar to the normal head CT data. This result also suggests transformers and segmentation networks with uncertainty may be used in tandem, with highly OOD images being filtered out by the transformer and the segmentation network providing meaningful uncertainty estimates on images that are only slightly OOD.

**Fig. 6:**
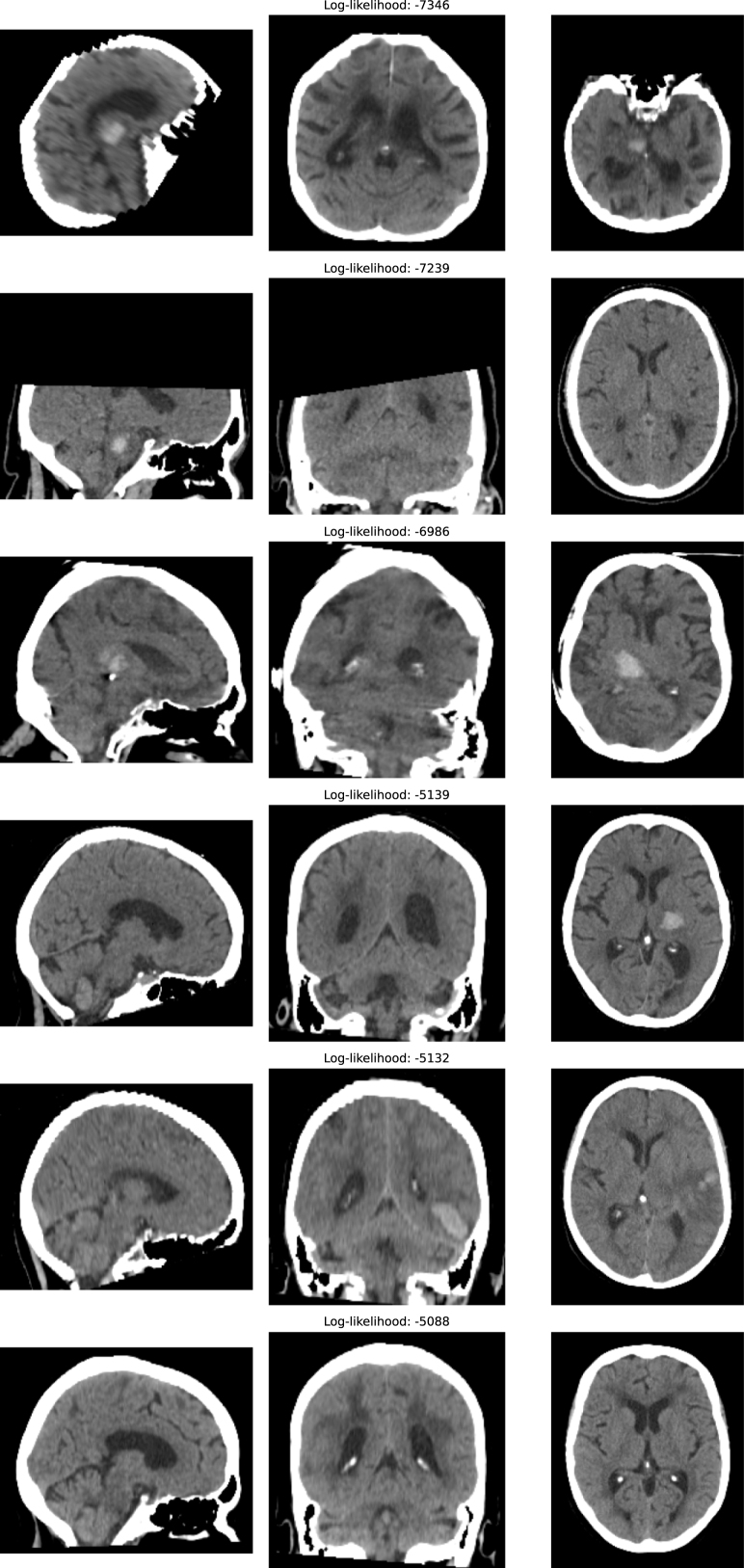
For the CROMIS dataset, this shows the three volumes assigned the lowest log-likelihood values (top three rows) and the highest values (bottom three rows), with three planes shown for each volume in columns 1-3: sagittal, axial, and coronal.

We evaluated the LTM on the CROMIS dataset in order to obtain some qualitative results on real data. While the CROMIS dataset is relatively clean, there are some low-quality scans. Fig. 6 shows the CROMIS volumes assigned the lowest and highest log-likelihood values. These results suggest the method is not only able to detect synthesised corruptions but is able to flag real-world data corruptions. The three lowest-likelihood scans showed a misregistration artefact, an FoV artefact causing the superior portion of the brain to be missing, and a motion artefact. It is worth noting the LTM was trained on CROMIS, and its ability to flag OOD scans in this dataset suggests the LTM is robust to having a small amount of anomalous data in the training set. This is encouraging as obtaining perfectly clean datasets for training is challenging in practice.

**Fig. 7:**
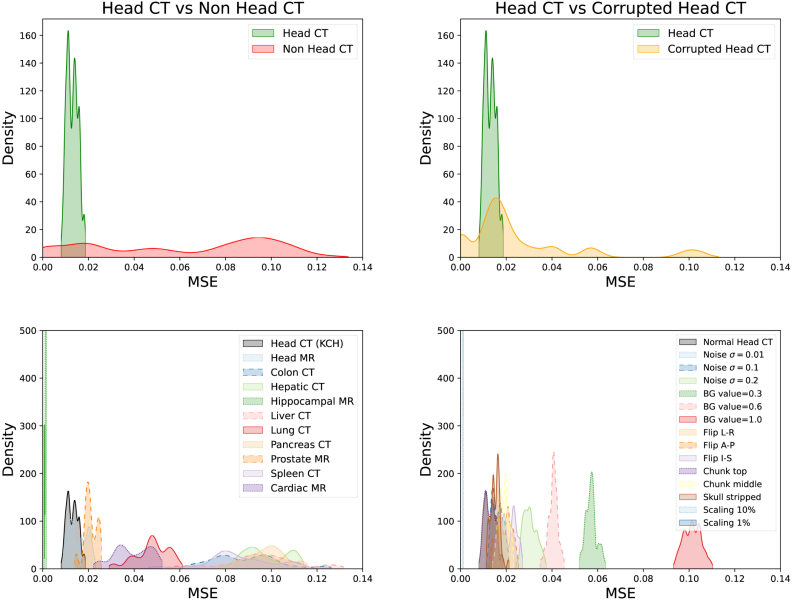
VQ-GAN reconstruction mean-squared error for the far-OOD case (left column) and the near-OOD case (right column), for coarse class labels (top row) and fine-grained labels (bottom row). These results show that reconstruction MSE alone is unable to identify OOD data.

We also investigated whether the VQ-VAE’s reconstruction error alone was sufficient to detect OOD data. We measured the mean-squared error (MSE) between the input and reconstruction, see Fig. 7. The results show substantial overlap between in-distribution and OOD MSE values, with the MSE for some classes much smaller than the MSE for in-distribution data. We also checked to see if this effect was driven by certain classes having overall darker intensity/more background than the CT images, by normalising each image’s MSE by its mean intensity. We found this improved class separation for the far-OOD case but not the near-OOD case, see Supplementary Fig 12. indicating the transformer component is essential for OOD detection. These results are in line with previous work finding that autoencoder-based reconstruction approaches tend to perform poorly at OOD detection (Yang et al., 2021, Salehi et al., 2021, Graham et al., 2023).

### 3.3 Ablation studies

We examined the effect of using a 3-layer rather than 4-layer VQ-VAE (which increases the size of the latent space by a factor of $2^{3}$), changing the loss function used during training to just the MSE or Perceptual loss, and removing the adversarial discriminator on the VQ-VAE reconstructions. Table 3 reports how these changes affected performance. A 3-layer model performed substantially worse than a 4-layer model; likely because the transformer found it harder to learn normality on the larger latent space. For 4-layer models, our results show that both adding the adversarial component and using a Perceptual loss help performance, but only when they are used in tandem. Adding a spectral component to the loss provides a modest performance increase over the perceptual loss used in the VQ-GAN.

**Table 3:**
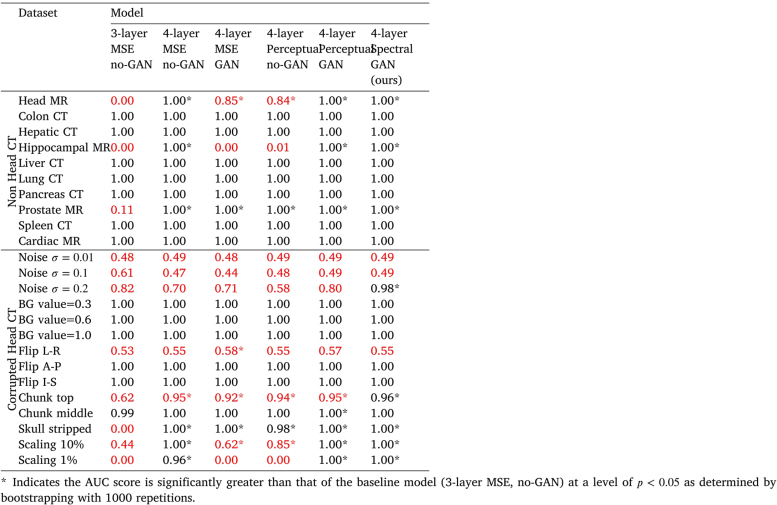
Study of changes to the VQ architecture and their influence on model performance. We report AUC scores for differentiating between each sub-class and normal CT data using the image log-likelihood values provided by each model (1.0 is perfect performance). Model elements changed are: layers: 3 or 4 layers in the encoder and decoder of the auto-encoder, losses: MSE − mean squared error, Perceptual − perceptual loss + MSE, Spectral − spectral + perceptual loss + MSE, GAN: whether or not there was an adversarial component to the training. Highlighted in red are any AUC scores <1.0 for the far-OOD non-head CT data, or ≤0.95 for the near-OOD corrupted head CT data.

**Fig. 8:**
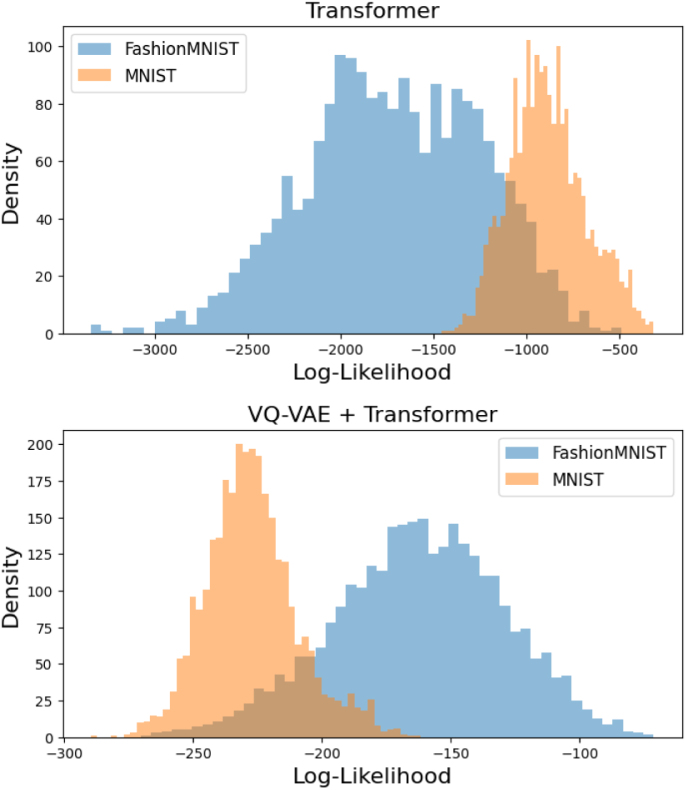
Log-likelihood on the FashionMNIST and MNIST test sets for models trained on FashionMNIST.

We also sought to investigate the impact of the VQ-VAE encoding on OOD performance by comparing likelihoods from a transformer on VQ-encoded data and on raw pixel data. It is not feasible to train transformers on raw pixel data of 3D data, so used 2D computer vision benchmarks. We selected FashionMNIST (Xiao et al., 2017) vs. MNIST (LeCun et al., 1998) as the in-distribution vs. OOD problem here. It has been widely reported that OOD detection is challenging on this dataset (Nalisnick et al., 2018, Choi et al., 2018, Hendrycks et al., 2018), and that models trained on FashionMNIST assign higher likelihoods to samples from MNIST. This result has been shown to hold true for VAEs, flow-based models, and PixelCNNs (Nalisnick et al., 2018). We trained two models on FashionMNIST: an LTM with a two-layer VQ-VAE (latent dimension 7 × 7), and a transformer directly on the pixel data (dimension 28 × 28). Fig. 8 shows the log-likelihoods evaluated on the test splits. The transformer model predicts higher log-likelihoods for MNIST, in keeping with findings for VAEs, flow-based models, and PixelCNNs. However, the LTM correctly assigned FashionMNIST samples higher likelihood than MNIST samples. This result suggests that the VQ-VAE’s role is not just providing a compressed representation of the data that makes transformer training tractable; it also provides a new representation of the data that can facilitate OOD detection. It has been suggested that failures of generative model-based methods can be due to the likelihood being dominated by image texture details rather than semantic content (Dieleman, 2020). These findings support this idea and suggest that working in a more abstract representation of the input can benefit OOD detection.

### 3.4 Anomaly maps

**Fig. 9:**
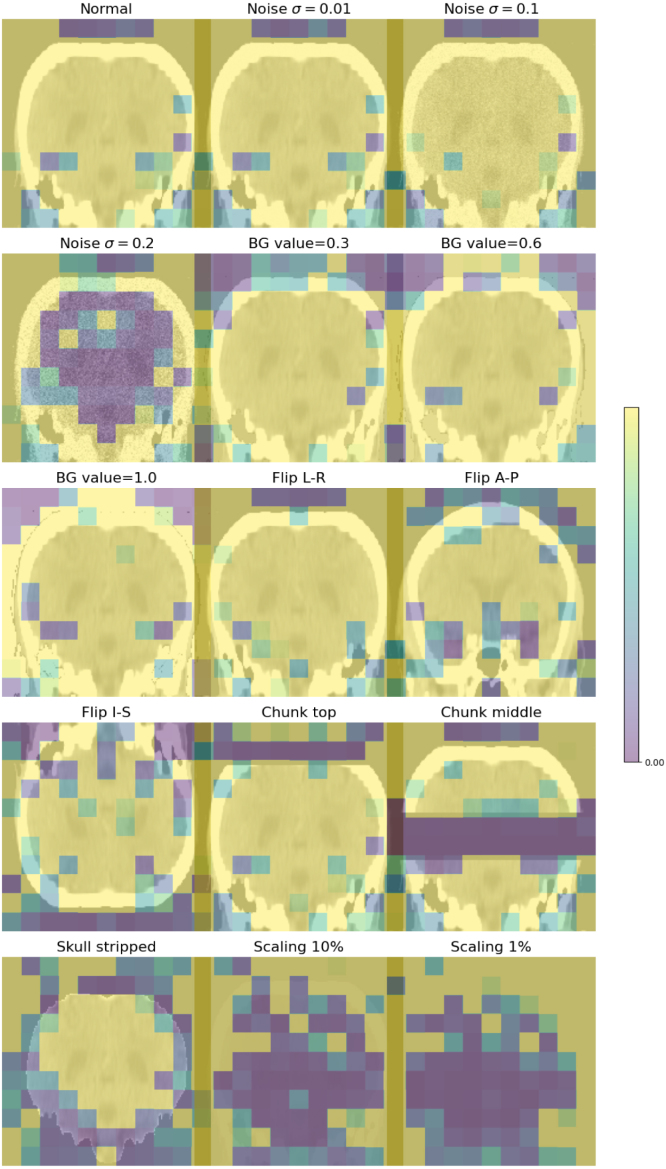
Spatial likelihood maps obtained from the transformer. The network accurately assigns low likelihood values to regions of corruptions, such as the brain in the noise σ=0.2 case, missing chunks, and the absent skull. The same images without likelihood values overlaid are shown in Fig. 1.

We investigate the ability of an LTM to localise image anomalies. These are calculated by taking the conditional probabilities for each code in the sequence, $p\left(s_{i}|s_{<i}\right)$ and reshaping back from 1D back to 3D. These conditional probabilities are then upsampled by a factor of 16 along each axis using nearest-neighbour interpolation to match the size of the latent representation to the size of the input image, allowing them to be directly overlayed. The results are shown in Fig. 9. The maps provide sensible localisation of artefacts, particularly in images with clear spatial anomalies: those with missing chunks or skull-stripped. The maps also highlight the neck and upper part of the skull in the image with interior-posterior flipping, and highlight everywhere the head should be in the images with intensity scaling. The maps also make clear that the model considers images with Gaussian noise at σ=0.2 to be OOD, clearly highlighting the brain tissue, but does not consider lower levels of noise to be OOD.

### 3.5 LTMs as a filter before segmentation

**Fig. 10:**
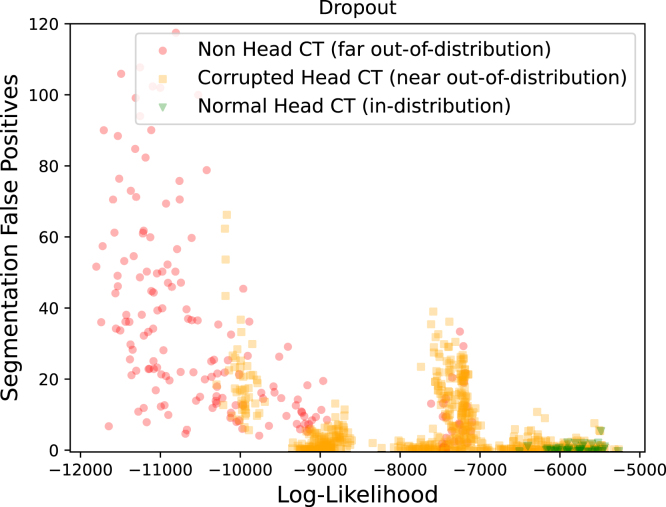
Image log-likelihood and the number of FP detections made by the dropout network.

To understand how an LTM might be used as a filter for OOD data before running downstream analysis tasks, we explored the relationship between an image’s likelihood and the performance of the segmentation network. We measured poor segmentation performance as the number of FP lesions predicted on a volume. Fig. 10 shows results for the best-performing dropout network and includes results for other networks, which are similar, in Supplementary Fig 11. The results indicate a strong relationship between the likelihood and the segmentation model’s ability to process images. The majority of high-FP segmentations could be filtered out by not running the segmentation network on images with a log-likelihood lower than −7000.

The results also show that corrupted CT-scans that were assigned similar likelihoods to the in-distribution data – images with low levels of noise or L-R flipped – tended to have a lower number of FP. This supports our claim that these are the subtlest of all the corruptions applied, and suggest it is not necessarily a problem that the LTM was not able to separate them from in-distribution data.

## 4 Discussion

In this work, we propose applying Latent Transformer Models to perform out-of-distribution detection on 3D medical data. These models use a VQ-VAE to encode an input into a semantically rich latent space of small enough dimension to allow for transformers to be trained on them. The trained transformer can be used to quantify the likelihood of a new sample, with samples being rejected as OOD using a one-sided threshold on this likelihood. This architecture has been used to enable high-resolution images synthesis in 2D (Esser et al., 2021) and 3D (Tudosiu et al., 2020, Tudosiu et al., 2022), and to perform unsupervised pathology detection (Pinaya et al., 2021, Pinaya et al., 2022), but, to our knowledge, this work is the first time they have been demonstrated for whole-image OOD.

Robustness to OOD data is crucial if we are to deploy deep-learning algorithms in the clinic, and such methods must be robust to both far-OOD and near-OOD data. The need to filter out far-OOD data may seem surprising, as it may be supposed to be straightforward to filter out images of a different modality using their DICOM tags in a clinical setting. However, our experience working with large clinical datasets reveals such datasets are messy enough that even DICOM tags can sometimes mislead.

It is commonly suggested that predictive methods that report their uncertainty can be used to create pipelines that are robust to OOD data. We tested three segmentation uncertainty methods and found that they catastrophically failed in the far-OOD domain; often making incorrect predictions with very high confidence. To our knowledge, such methods have only been tested on near-OOD data before (Haas and Rabus, 2021, McClure et al., 2019), and this work represents the first time that segmentation uncertainty methods have been tested in the far-OOD domain. Our results show these methods cannot be replied upon to provide robustness to far-OOD data.

The failures of task-specific uncertainty methods motivate the use of a filter to ensure that only in-distribution data reaches our predictive networks. Our results demonstrate that the LTM effectively identifies OOD scans, in both the far-OOD and near-OOD domains. Whilst identifying far-OOD data may appear to be a simple task for generative models, results on 2D computer vision datasets have shown these models can fail catastrophically, assigning higher likelihoods to far-OOD than in-distribution images (Nalisnick et al., 2018, Choi et al., 2018), so it is reassuring to verify out method achieved perfect identification on 15 different far-OOD datasets. We showed that a transformer operating on directly on pixel data also fails catastrophically on computer vision datasets, but the LTM does not, suggesting that the VQ-VAE plays an important role in the LTM’s OOD performance. It is possible that it helps by abstracting away some of the low-level image details, allowing the transformer to focus on the more relevant high-level content.

There are several advantages to an LTM-based filter for OOD detection. Firstly, unlike task-based models with uncertainty, this class of models can be trained in a fully unsupervised way. The only requirement is that the training dataset represents non-anomalous data. However, analysis of the training dataset itself, using the trained LTM, revealed a small number of anomalous scans in the training data, indicating the LTM is robust to such degree of contamination from anomalous training data. Another benefit of using a generative OOD model as a filter is that the model is not tied to the downstream task that is being performed. One could imagine a multi-model pipeline that performs several tasks, for example, one that aims to quantify brain volume and segment lesions. The algorithms that perform each of these tasks may have different tolerances for image quality, and using a filter that provides a continuous likelihood score allows for different thresholds to be set for the downstream task networks, depending on their tolerance for anomalous data.

Our method was further able to provide spatial maps that highlight the region of the image the transformer considers to be OOD. Such maps can both facilitate understanding of why an image was rejected as OOD and help to increase confidence that the model decisions are being made for the right ‘reasons’. They could also be useful in allowing downstream networks to be run on the images but having results from certain parts of the image discounted — for exampling, ruling out any lesions detected in a portion of the brain with severe movement artefacts whilst still allowing for lesions to be detected in other, artefact-free regions.

We performed an ablation analysis to understand which features were critical to model performance. We found that the level of VQ-VAE compression is crucial; at lower levels performance is much poorer. We also found using perceptual and adversarial losses improved performance, suggesting that losses designed to improve the quality of reconstructions in turn improve the quality of the latent representation in a way that is useful for OOD detection.

Our results show that uncertainty methods are better able to rule out poor predictions when the data is near-OOD, suggesting that a combination of OOD filters and predictive uncertainty can be used in tandem to further robustify pipeline, with the filter used to remove very OOD data and uncertainty methods giving a useful measure of confidence for near-OOD cases.

## 5 Conclusion

In this work, we consider how to mitigate the effect of OOD data on clinically deployable image-processing pipelines, considering both near- and far-OOD data. We consider the specific task of segmenting lesions in 3D head CT data. We show that segmentation methods that provide uncertainty measures are not robust to OOD data, in particular failing catastrophically for far-OOD data. We propose the use of an LTM to filter OOD data and show the network can flag both near- and far-OOD data. The LTM is further able to provide spatial maps to highlight OOD regions. We believe this is one of the first applications of generative models to perform fully 3D, unsupervised OOD detection.

## Declaration of competing interest

The authors declare the following financial interests/personal relationships which may be considered as potential competing interests: Mark Graham, Paul Wright, Walter Diaz Sanz, Parashkev Nachev, Sebastian Ourselin, Geraint Rees reports financial support was provided by Wellcome Trust. Yee Mah reports financial support was provided by UKRI Medical Research Council.

## Data Availability

The data that has been used is confidential.

## Appendix A Supplementary data

MMC S1 Supplementary figures.

## Footnotes

1 https://monai.io/.
