# Supplementary material for: Latent Transformer Models for out-of-distribution detection
Source: Med Image Anal. 2023 Dec;90:102967. doi: 10.1016/j.media.2023.102967 (PMC10900071; doi:10.1016/j.media.2023.102967)
Supplement: MMC S1 — Supplementary figures. [file mmc1.pdf]

Fig. 11. Image log-likelihood and the number of FP detections made by the baseline and ensemble networks.

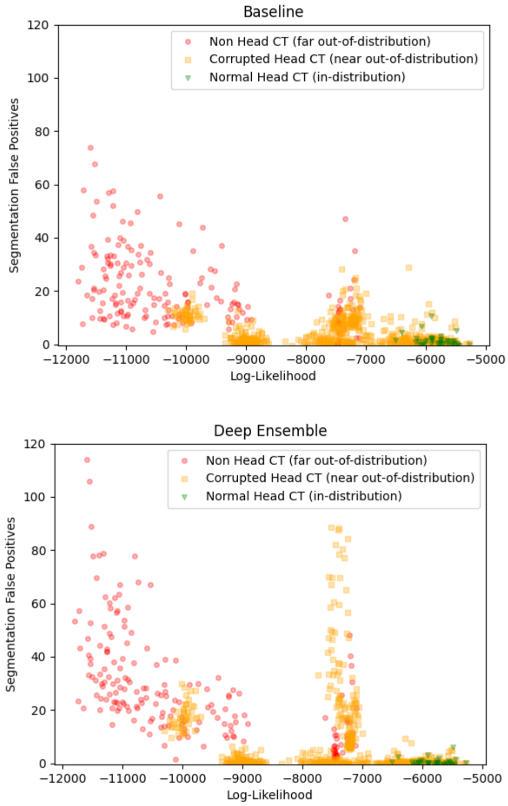

Supplementary Material

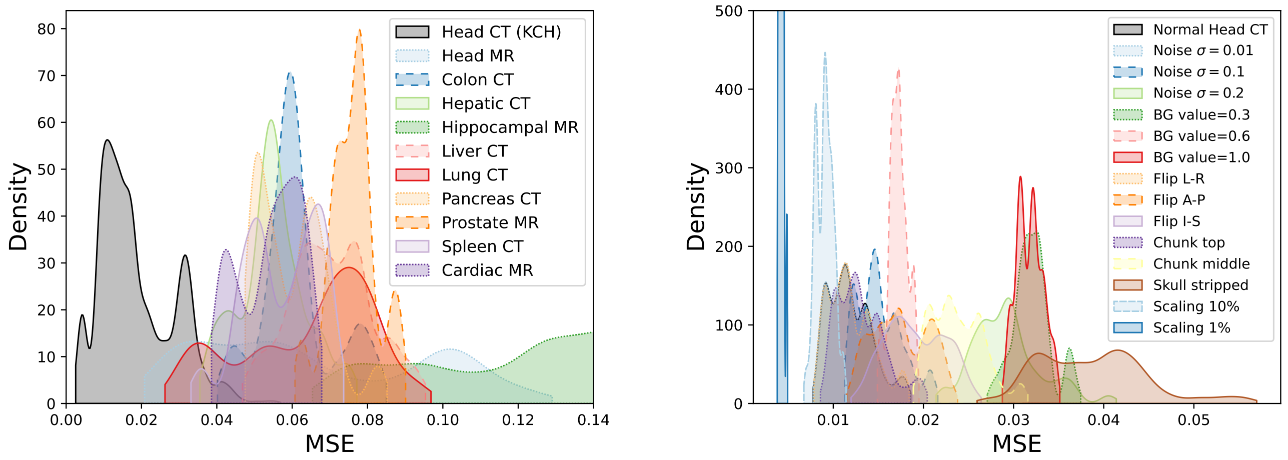

Fig. 12. VQ-GAN reconstruction mean-squared error for the far-OOD case (left) and the near-OOD case (right), with normalisation for the mean intensity of each image.
